# Application of biomaterials in mesenchymal stem cell based endometrial reconstruction: current status and challenges

**DOI:** 10.3389/fbioe.2025.1518398

**Published:** 2025-01-29

**Authors:** Ling He, Qianrong Li

**Affiliations:** Hospital of Chengdu University of Traditional Chinese Medicine, Chengdu, Sichuan, China

**Keywords:** biomaterials, mesenchymal stem cell based therapy, endometrial reconstruction, thin endometrium, intrauterine adhesion

## Abstract

Severe endometrial injuries may cause thin endometrium and intrauterine adhesion in women which can result in uterine factor infertility. Current treatments, including surgical separation of adhesions and hormonal regeneration of the endometrium, often fail to prevent re-adhesion and achieve satisfactory reproductive results. Recently, mesenchymal stem cells (MSCs) have become a promising new treatment for IUA. However, challenges such as cell survival and transplantation limit the effectiveness of MSC therapy. Researchers have explored various approaches to enhance the therapeutic efficiency of MSCs. Among these, biomaterials have been frequently employed due to their biocompatibility, degradability, and ability to provide a conducive environment for cell growth. This review discusses the use of various biomaterials in MSC-based therapies for endometrial reconstruction and summarizes evidence from preclinical and clinical studies, highlighting the efficacy and safety of these biomaterials. The review also addresses future directions in this field, such as advances in biomaterial engineering, new biomaterials currently under investigation, and personalized medicine approaches. This review emphasizes the significance of biomaterials in MSC-based therapy for endometrial reconstruction and provides practical guidance for developing new materials and treatment protocols for clinical applications.

## 1 Introduction

As the place for embryo implantation, the endometrium plays a crucial role in human reproduction. Severe injuries of endometrium can lead to thin endometrium and intrauterine adhesions in women which may result in fertility (Foix et al., 1966; Lee et al., 2021). Consequently, with a crucial role in restoring the normal function of the uterus, endometrial regeneration has been paid more and more attention in the treatment of endometrium-related infertility. Currently, the traditional treatment, encompassing transcervical resection of adhesions (TCRA) and physical barriers that create spatial conditions conducive for endometrial regeneration, hormonal therapy and other agents that promote endometrial proliferation, which establish an optimal internal environment for endometrial healing, have been implemented in clinical practice (AAGL Advancing Minimally Invasive Gynecology Worldwide, 2010). However, these traditional therapeutic strategies fail avert the re-adhesion and satisfactory fertility outcome (Capella-Allouc et al., 1999). Promising new alternatives, such as cell transplantation including mesenchymal stem cells (MSCs) transplantation have emerged (Tan et al., 2016; Santamaria et al., 2016). Although the therapeutic effect and translational value of MSCs has been tested in both animal models and patients, significant challenges still remain.

Poor cell survival can significantly limit the efficacy of MSC-based therapies, as the therapeutic benefits of MSCs are closely tied to their ability to engraft, proliferate, and exert paracrine effects at the site of injury. Biomaterials with three-dimensional (3D) architecture can serve as physical barriers to prevent adhesion formation, while also offering a docking site for MSCs to facilitate the regeneration of the endometrium. Furthermore, biomaterials can be applied in 3D bio-printing to construct artificial endometrium that can be used to repair severely damaged endometrium. In the meantime, other MSC-related therapies can be performed with biomaterials too. Additionally, to reduce the ethical issues brought by cell transplantation, biomaterials were applied as carriers of MSC based secretomes, exosomes and apoptosis bodies in endometrial regeneration.

In summary, the integration of biomaterials into the MSCs based endometrial regeneration therapy represents a promising Frontier in reproductive and regenerative medicine. The biocompatibility and degradability of biomaterials not only ensured minimal provocation of adverse reactions and reduced extra endometrial damage from invasive procedures. By leveraging these unique properties of biomaterials, researchers are developing new and effective treatments that can promote endometrial regeneration and reproductive outcomes. In this review, we explored the various types of biomaterials used in MSCs based endometrial regeneration, highlighting their potential to revolutionize treatment approaches and improve the quality of life for affected women.

## 2 Traditional therapeutic strategies of endometrial regeneration

Severe injuries of endometrium induced by trauma, surgery or infection can lead to thin endometrium and IUA based infertility (Foix et al., 1966; Lee et al., 2021). In particular, adhesions physically reduce the functional endometrial surface area, impairing the ability of the endometrium to proliferate and thicken adequately during the menstrual cycle. Furthermore, the Insufficient endometrial thickness can hinder embryo implantation and development, thereby increasing the risk of infertility or recurrent miscarriage. These changes can compromise reproductive function and contribute to fertility challenges for affected individuals (Asherman, 1948; Bosteels et al., 2017). IUA patients with successful pregnancies may experience symptoms like placental adhesion, implantation issues, and premature delivery (Schenker and Margalioth, 1982). These clinical symptoms significantly impact the menstrual health and fertility of women of childbearing age. Consequently, with a crucial role in restoring the normal function of the uterus, endometrial regeneration has been paid more and more attention in the treatment of endometrium-related infertility.

Traditional therapeutic strategies for IUA primarily focus on surgical and supportive interventions aimed at restoring the uterine cavity’s normal architecture and function. The most common approach is direct hysteroscopic visualization and transcervical resection of adhesion which can immediately remove internal adhesions and reopen the uterine cavity (AAGL Advancing Minimally Invasive Gynecology Worldwide, 2010). However, the ideal result for IUA therapy is to boost fertility by restoring the damaged endometrium. TCRA can only achieve an immediate reduction of internal adhesions and increased pregnancy rates in a short time (Rein et al., 2011). In long-term follow-up visits study, approximately 60% of severe IUA cases recur after TCRA and leads to infertility (Capella-Allouc et al., 1999). To prevent IUA re-forming, TCRA is often followed by the insertion of physical barriers, such as intrauterine devices (IUDs), Foley catheters or intrauterine balloons, to prevent reformation of adhesions during the healing process (Orhue et al., 2003; Shi et al., 2019). These physical barriers have shown promise in reducing the recurrence of adhesions, but their efficacy in improving long-term reproductive outcomes remains suboptimal (Gupta et al., 2013; Salma et al., 2014). The commonly used intrauterine physical barriers are usually non-biodegradable and require surgical placement and removement. Thus, the use of these barriers may cause inflammatory reactions and endometrium compression which may derive amenorrhea and result in inhibition of endometrial regeneration (Orhue et al., 2003; Dreisler and Kjer, 2019).

In the realm of reproductive medicine, endometrial thickness is a crucial factor influencing embryo implantation (Shaulov et al., 2020). Hormone therapy, particularly the administration of estrogen, has shown significant benefits to promote endometrial thickness (Johary et al., 2014). However, it is important to note that traditional hormone-based treatments typically involve the use of high doses of estrogen, which can unfortunately lead to a range of prolonged side effects, including serious complications such as clotting disorders, which may pose additional health risks to the patients undergoing such therapies (AAGL Advancing Minimally Invasive Gynecology Worldwide, 2010; Canonico et al., 2008).

In summary, these traditional therapies are often limited by high recurrence rates and side effects particularly in cases of severe adhesions, and incomplete recovery of endometrial function. These limitations have prompted ongoing research into alternative strategies, especially stem cell therapy, to enhance the regeneration of endometrium.

## 3 Therapeutic potential of mesenchymal stem cells in endometrial regeneration

After being initially presented by Prianishnikov (1978), the fascinating concept of endometrial stem cells (EnSCs) was ultimately isolated and characterized by Chan and his colleagues in 2004 Chan et al. (2004). Following this groundbreaking discovery, Gargett and colleagues further confirmed the crucial role that EnSCs play in the processes of endometrial proliferation and the healing of injuries within the uterine lining, highlighting their importance in female reproductive health (Gargett et al., 2016). With the advancements in single-cell transcriptomics and single-molecule fluorescence *in situ* hybridization imaging, Marečková et al. achieved a significant breakthrough by successfully identifying a distinct population of CDH2+ EnSCs located within the basalis (Marečková et al., 2024) Characterizing the transcriptomic profile of the EnSCs unveils new pathways for investigating their function in endometrial repair and regeneration, alongside disease pathophysiology. Currently, researchers hypothesized that reduced endometrial stem cells and damaged endometrial microenvironment may be responsible for endometrial associated infertility (Xu et al., 2020). Therefore, the application of stem cells as a therapeutic approach to treat endometrial injury has emerged as a potentially effective strategy (Gao et al., 2021).

MSCs have emerged as a promising therapeutic option in endometrial regeneration due to their regenerative and immunomodulatory properties. Firstly, studies have shown that MSCs can differentiate into endometrial-like epithelial and smooth muscle tissues (Arezoo et al., 2021; Hua et al., 2022). However, some recent studies have claimed that the regeneration effect of MSCs is more reliant on the paracrine effect than on a direct conversion into functional endometrial cell (Wang et al., 2014; Huang et al., 2024a; Park et al., 2018; Li et al., 2019a). The MSCs can stimulate cell proliferation, suppress fibrosis, and regulate the immune system through signaling factors, such as MicroRNA, cytokines, released either directly (Park et al., 2018; Zhang et al., 2022a) or via exosomes (Bonavina et al., 2024; Yao et al., 2019). Given these diverse functions, MSCs have been the focus of extensive research and application within the realm of endometrial regeneration. The therapeutic potential of several MSCs from different sources have been administrated in endometrium regeneration, including bone marrow MSCs (BMSCs) (Gao et al., 2019; Nagori et al., 2011; Zhao et al., 2015; Jing et al., 2014), menstrual blood derived MSCs (MenSCs) (Tan et al., 2016; Zhang et al., 2019), umbilical cord derived MSCs (UC-MSC) (Zhang et al., 2022a; Zhang et al., 2018) and adipose tissue derived MSCs (AD-MSCs) (Kilic et al., 2014).

**FIGURE 1:**
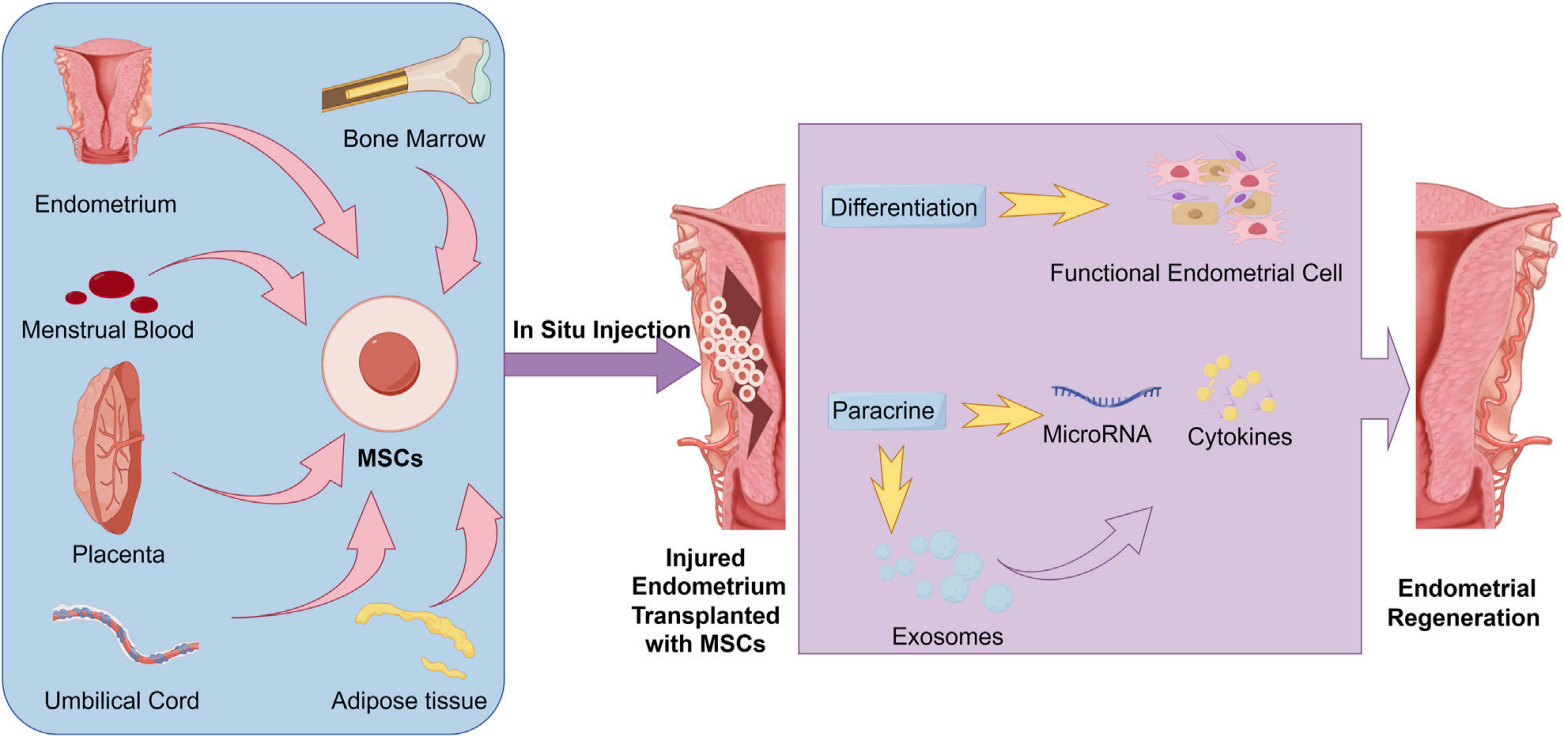
Common strategies and potential mechanisms of MSC transplantation in Endometrial regeneration.

Nevertheless, several researches have indicated that insufficient quantities and survival time of transplanted MSCs in injury site may result in unsuccessful tissue regeneration (Passier et al., 2008). Thus, in MSCs based endometrial regeneration therapy, to improve the number of MSCs delivered to the site of endometrium, *in situ* cell suspensions injection such as intrauterine injection (Gan et al., 2017; Huang et al., 2022a), intraperitoneal injection (Zheng et al., 2020) and spiral arterioles delivery by catheterization (Santamaria et al., 2016) were commonly utilized for MSCs delivery. These MSC therapeutic strategies used in endometrial regeneration and the potential mechanisms have been represented in detail in Figure 1.

However, the fluid shear stress induced by directly injection of cell suspensions often leads to limited survival rate of transplanted cells in target tissue (Ullah et al., 2019; La, 2012). Therefore, the extremely low cell survival rate and uneven distribution of cells after injection have been the major obstacles for MSC transplantation in endometrial regeneration (Zheng et al., 2020; Alawadhi et al., 2014; Cervelló et al., 2015). Biomaterials with good biocompatibility and 3D structure may create an effective approaches to increase cell retention and survival (Azizi et al., 2018). To address these limitations, biomaterials, such as hydrogels and collagen scaffolds, have been utilized to improve the survival and distribution of MSCs at the site of injury. These materials provide a supportive environment that enhances MSC retention, promotes cell proliferation, and facilitates the controlled release of therapeutic factors. Nevertheless, the immunogenicity, potential tumorigenicity and teratogenicity of transplanted MSCs are still greatly challenged their clinical practice (Heslop et al., 2015; Benor et al., 2020). Thus, endogenous MSCs or MSC-derived secretome, especially extracellular vesicles, has attracted more and more attentions (Park et al., 2018; Li and Hu, 2023).

In summary, although MSCs possess remarkable promise for endometrial regeneration, there are also considerable obstacles. Surmounting these challenges through the application of sophisticated biomaterials is crucial to enhance its therapeutic effectiveness and guarantee safe clinical application.

## 4 Biomaterials used in MSCs delivery of endometrial regeneration

**FIGURE 2:**
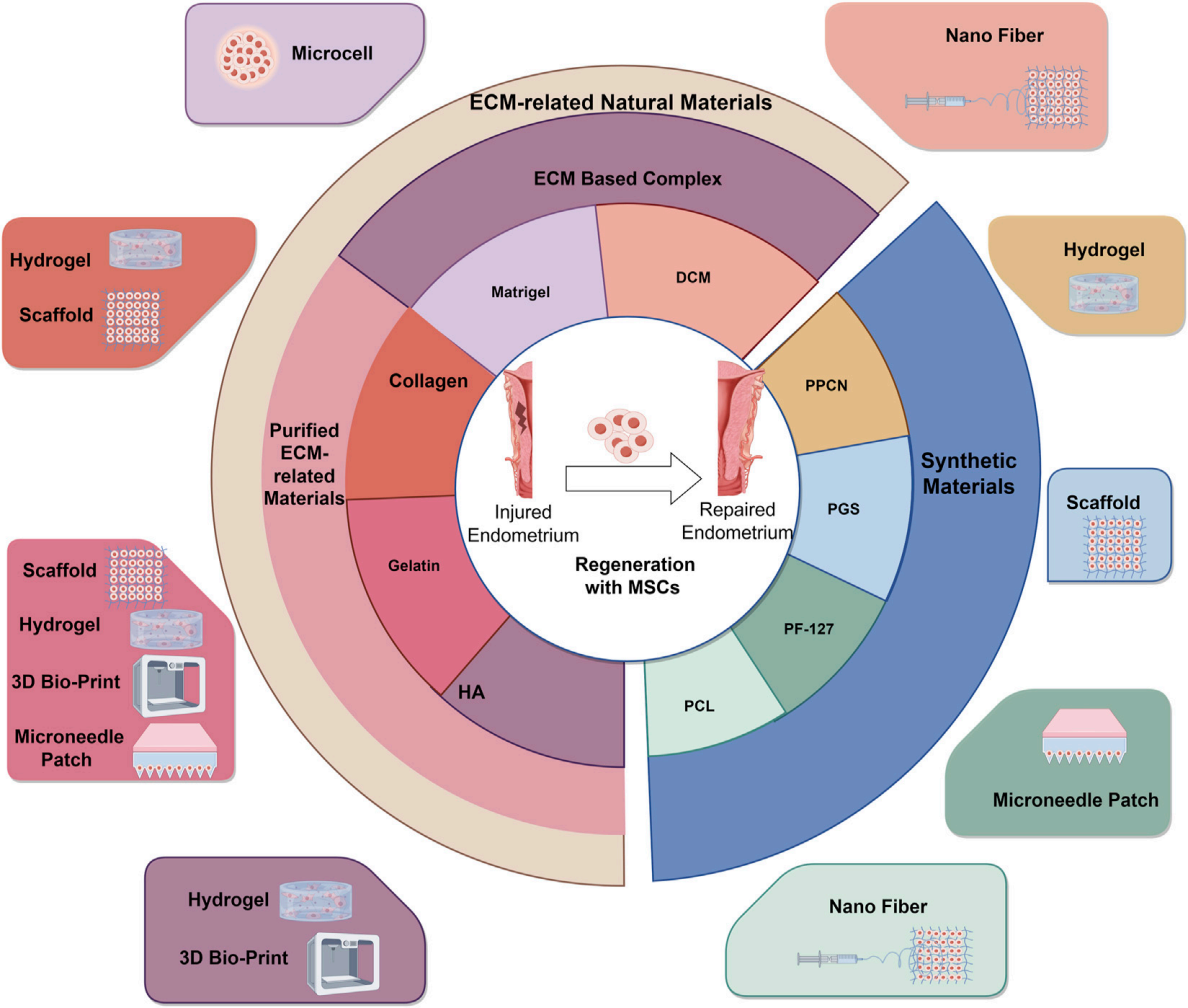
Biomaterials based MSCs Delivering in Endometrial Regeneration.

As mentioned above, one of the critical challenges in MSC based therapy is maintain the biological activity of transplanted MSCs in the injury tissues (Levy et al., 2020). However, the poor MSCs viability after cell suspension injection has been proved to limit fertility elevation in IUA therapy (Song et al., 2021). As the extracellular matrix (ECM) can supply bioactive factors, 3D structural support, and morphological guidance to the cells (Jian et al., 2021a), loss of anchorage-dependent attachment to the ECM has been reported to be a key reason of cell apoptosis (Frisch and Francis, 1994). In recent years, incorporating ECM based natural biomaterials and/or synthetic biomaterials with ECM-like properties with MSCs have been administrated to address this problem and made significant progress. Consequently, in this review, we summarized both natural and synthetic materials that are utilized for MSC based endometrial regeneration as shown in Figure 2.

### 4.1 ECM-related natural materials

The RGD domain (Arg-Gly-Asp) is a well-known integrin-binding site that facilitates cell adhesion, migration and signal transduction in the tissue repair process. ECM based natural materials containing the RGD motif, such as **Decellularized Matrix (DCM), Matrigel, collagen and gelatin**, played a critical role in enhancing MSC adhesion and survival. Other non-protein ECM based materials, such as Hyaluronic acid (HA) a glycosaminoglycan, although without RGDs, still valued for its remarkable biocompatibility and anti-inflammatory properties (Nappi et al., 2007). These natural materials offer several advantages, including excellent biocompatibility, bioactivity, and the ability to closely mimic the native ECM environment, which is crucial for supporting MSC function in endometrial regeneration.

#### 4.1.1 ECM based complex in MSCs delivery

**Decellularized Matrix (DCM)** are tissues from which cells have been removed, leaving behind an intact ECM scaffold. DCM used in regenerative medicine can provide a biomimetic nutrient environment and 3D-structure for cell proliferate and attachment with minimized immune response following implantation (Taylor et al., 2018; Aamodt and Grainger, 2016; Shakouri-Motlagh et al., 2017). For example, when co-cultured with smooth muscle cells in a decellularized endometrium, human MSCs can differentiate into endometrial epithelial and stromal cells and finally rise endometrial-like tissues *in vitro* (Sargazi et al., 2023). Arezoo et al. tested the *in vitro* culture efficiency of MenSCs in the uterine derived DCM and observed MenSCs nesting in the natural pore of uterine (Arezoo et al., 2021). Their study brought us forward possibilities for the ultimate goal of bioengineering the entire human womb. Thus, DCM-derived biomaterials have become competitive and safe alternatives for IUA treatment in both preclinical and clinical researches.

In MSCs based endometrial regeneration, DCM derived from different sources have been administrated (Miyazaki and Maruyama, 2014; Li et al., 2021; Ji et al., 2022). As DCM retains its specific functions and biochemical/structural compositions from original tissue (Campo et al., 2019), endometrium derived DCM have been the most widely used DCM in endometrial regeneration. After transplanted with rat (Miyazaki and Maruyama, 2014) or human (Li et al., 2021) MSCs re-cellulated DUM, the partially excised rat uteri demonstrated recellularization and regeneration of uterine tissues, achieving pregnancies nearly comparable to those in intact uteri.

Based on the exploring in DCM studies, other complex materials which are enriched with ECM components, for example, Matrigel and other extracellular matrix-derived nanofibers or films, have been utilized in endometrial restoration. Zhu et al. developed a human-chorionic-villi (CV)-derived nanofiber-based 3D MSC culture system. The CV-nanofibers maintained the characters of CV matrix and achieved large-scale culture of MSCs and enhanced EV secretion. Based on this, they further encapsulated MSC derived EVs into nanofibers and achieved promoted endometrial regeneration and facilitated fertility restoration by transplanting of them into rat IUA models (Zhu et al., 2023).

**Matrigel** is a commercial native extracellular matrix-like material derived from the Engelbreth-Holm-Swarm mouse sarcoma (Kleinman and Martin, 2005). With the ability to mimic the complex extracellular environment found in many tissues, Matrigel were widely used in in vitro 3D cell culture. By seeding HEMSC derived endometrial epithelial progenitor cells (EEPCs) in Matrigel, Jiang et al. established endometrial membrane organoids (EMOs) *in vitro*. Then injection of these Matrigel enrolled EMOs successfully improved scar recovery in IUA rat model (Jiang et al., 2021b). Xu et al. prepared monodisperse UCMSC-loaded/Matrigel microspheres with a microfluidics device which can offer higher cell retention *in situ* and help to promote regeneration, neovascularization, and recovery of function in rat IUA mode (Xu et al., 2021).

In summary, delivering MSCs through ECM-based complexes poses significant challenges, including high costs and variability between batches. Due to these issues, there is an increasing preference among researchers for using purified ECM-related molecules like collagen, gelatin, and hyaluronic acid to offer more consistent and reliable quality for therapeutic applications. This shift is expected to greatly improve the biocompatibility and therapeutic effectiveness of MSC based treatments, leading to better clinical outcomes.

#### 4.1.2 Purified ECM-related materials in MSCs delivery

**Collagen** is the most abundant protein in the ECM. The good biocompatibility and biodegradability of collagen have made it a competitive candidate to support cell adhesion, migration, and tissue structural regeneration (Cao et al., 2018; Heino, 2007). Regarding its physical characteristics, the rigidity of crosslinking collagen scaffolds makes them ideal for maintaining the physical architecture of the damaged uterine tissue, which is critical in cases of severe adhesions. Besides to be as physical barriers, as a part of ECM, collagen offers the potential to incorporate growth factors or other bioactive molecules which can be released over time and provide a highly advantageous platform for loading and delivering MSCs. The sizes controllable pore structure can facilitate the migration and distribution of MSCs, as well as the diffusion of nutrients and oxygen (Ferreira et al., 2012). Additionally, one of the most significant advantages of using collagen scaffold to deliver MSCs is the straightforward method of preparation. Gently dripping the MSCs onto the scaffold can minimize mechanical stress on the cells, preserving their viability and functional properties. Thus, crosslinked collagen scaffold is more commonly used in MSCs based endometrium repair in both patients and animal models.

In the preliminary MSC based therapy, AD-MSC formed scaffold-free 3D cell sheets have been used for rat endometrium regeneration and resulted in improved pregnancy outcomes (Sun et al., 2018). Nonetheless, the intricate cultural procedures have significantly restricted the application of MSC patches. Dai et al. constructed an AD-MSCs loaded collagen porous scaffold (CS/AD-MSC), which resulted in increased endometrial thickness and gland formation while effectively mitigating fibrosis in rat IUA model (Dai et al., 2023). A 3D bioprinting collagen endometrial patch loaded AD-MSCs has been developed by Hong et al. and received better fertility outcome in rat thin endometrium model (Hong et al., 2023). Moreover, Xin et al. developed BMSCs (Ding et al., 2014) and UC-MSCs (Xin et al., 2019) loaded collagen scaffolds respectively. Transplantation of both these two MSC loaded collagen scaffolds into uterine cavity can promote endometrial regeneration and fertility restoration in rat IUA model. In addition, the collagen scaffold loaded with human UC-MSCs significantly regulated the expression levels of fibrosis, estrogen, and differentiation-related genes in rat endometrium damage model (Liu et al., 2020). Moreover, loaded with human embryonic stem cells (hESCs) derived endometrium-like cells, collagen scaffolds successfully retrieve the structure and function of uterine horns in a rat model of severe uterine damage and limited the uncontrolled stem cell differentiation *in vivo* (Song et al., 2015).

In the human endometrial tissue, CD146+ PDGFRβ+ human endometrial perivascular cells (En-PSCs) were defined as endometrium stem cells. With artificially over expressed cysteine-rich angiogenic inducer 61 (CYR61) to promote vascular development, CYR61 overexpressed En-PSCs were incorporated into collagen scaffolds and achieved enhanced regeneration of both the endometrial and myometrial tissues, as well as the promotion of neovascularization in the uteri of rats (Li et al., 2019b). Compared to other endometrial stem cells, MenSCs which are origin from menstrual blood are notably easier to obtain, as they can be collected non-invasively from menstrual flow (Liu et al., 2018). Hu et al. applied human MenSCs loaded collagen scaffold in long term rat IUA model and successfully promoted endometrium regeneration (Hu et al., 2022a).

Collagen scaffolds infused with MSCs show great promise for improving the repair of damaged endometrial tissue, presenting a valuable option for therapeutic intervention. Notably, MSCs derived from umbilical cord (UCMSC) have been loaded collagen scaffolds and progressed to phase I clinical trials, marking a significant advancement in their use within regenerative medicine (Cao et al., 2018; Zhang et al., 2021). In the clinical trial NCT03724617, the UC-MSCs loaded collagen scaffold has been recognized as a promising and innovative method for treating women with unresponsive thin endometrium, demonstrating its potential to tackle a difficult issue in reproductive health (Zhang et al., 2021). Additionally, the safety and effectiveness of MSC-loaded collagen scaffolds have been thoroughly assessed in clinical trial NCT02313415, focusing on patients who suffer from recurrent IUA after surgery, underscoring the critical nature of this research in enhancing patient outcomes (Cao et al., 2018). Moreover, MSC-loaded collagen scaffolds have been tested in various other clinical trials, although the findings from these studies have yet to produce definitive clinical results. It still highlighted the ongoing necessity for further exploration in this promising area.

**Gelatin** is a protein obtained through the partial hydrolysis of collagen. Due to the absence of aromatic free radicals, gelatin show a better biocompatibility than collagen (Gorgieva and Kokol, 2011). Furthermore, by maintaining bioactive sequences derived from collagen, including RGD motifs and MMP-9 degradation sites, gelatin preserves the capacity for cellular adhesion akin to that of collagen (Vandooren et al., 2013). However, gelatin demonstrates instability and displays inadequate mechanical characteristics at physiological temperature, which limited the administration in clinic. Thus, by incorporating sodium alginate to enhance gel properties, Ji et al. successfully developed a gelatin hydrogel scaffold which was loaded with human induced pluripotent stem cell-derived MSCs (hiMSCs). This innovative scaffold created using 3D bioprinting featured a meticulously designed porous grid-like structure which can improve cellular integration and functionality (Ji et al., 2020).

Linking methacryloyl groups on gelatin forms **Gelatin methacryloyl (GelMA)**. The gelatin in GelMA retains the biocompatibility derived from collagen, while the methacryloyl groups allow GelMA to be photo-crosslinked, allowing it to produce stable hydrogels with adjustable mechanical characteristics. Thus, GelMA has been widely used in tissue engineering due to its favorable properties that combine the natural benefits of gelatin with the versatility of synthetic materials (Kurian et al., 2022).

Lu et al. developed injectable porous scaffolds for the delivery of AD-MSCs using microfluidic 3D bioprinting with a GelMA/PEO (polyethylene oxide) bioink (Lu et al., 2023). During the 3D bioprinting process, UV irradiation of suitable intensity was applied to solidify the GelMA microfibers. The scaffold was constructed through layer-by-layer stacking of the GelMA microfibers, followed by complete UV exposure. After dissolving the PEO droplets in aqueous solutions, controllable micropores were formed, which may facilitate the encapsulation and survival of AD-MSCs within the scaffold. Following *in situ* injection into the injured endometrium of IUA rats, these AD-MSC loaded GelMA-based scaffolds can augment functional endometrial regeneration by mitigating the inflammatory response, fostering cell proliferation, and enhancing vascularization.

By adding methacrylated collagen (ColMA) into GelMA hydrogel, Feng et al. achieved tissue hydrogels with appropriate swelling ratio, enhanced mechanical properties, and impressive stability. Loading this hydrogel with amniotic MSCs (AMSCs) by 3D biological printing technology, they realized slow release of AMSCs and successfully prevented cavity adhesion in a rat IUA model (Feng et al., 2021). In MSC-based therapy, sericin, a main component of natural silk, has been reported to enhance cell adhesion and proliferation with antioxidant properties (Chouhan and Mandal, 2020). Chen et al. developed an injectable hydrogel with GelMA and methacrylate sericin (SerMA) matrix to delivery human UC-MSC in a rat IUA model. The interpenetrating polymer network (IPN) structure, formed by these two materials through UV exposure, allows MSC cells to integrate into the damaged tissue and aid in tissue repair (Chen et al., 2023).

CD34^+^ human endometrium-derived adventitial cells (En-ADVs) have been identified as a kind of endometrium stem cells with a promising degree of plasticity and to be able to differentiate into vascular endothelial-like cells and endometrial stroma-like cells (Zhu et al., 2021). Thus En-ADVs have been taken as innate progenitors of MSCs in the uterus and efficient seed cells for tissue regeneration. As 3D cell spheroids displayed higher potential for cell proliferation, differentiation, and migration than dissociated cells (Griffin et al., 2022), 3D En-ADVs spheroids have been regarded as potential candidates for endometrium regeneration. Nonetheless, the intricacy of 3D En-ADVs spheroid culture extremely limited its application (Domnina et al., 2018). Li et al. designed an En-ADVs spheroid-loaded antimicrobial flexible microneedles (MN) with GelMA. After curried with UV light, the microwells formed in MN provided a place for En-ADVs gathering and further spheroid culturing. After cultured En-ADVs in microwells, the En-ADVs spheroids were formed in MN. Then MN patch coated with Matrigel was adjusted to the intrauterine surface and release 3D En-ADVs spheroids directly to the injured sites. With the application of MN/En-ADV, the repaired uteri show well-defined myometrial regeneration, angiogenesis, and an increase of endometrial receptivity in a rat model (Li et al., 2022).

As perivascular cells are postulated to be the precursors of tissue-specific MSCs (Yianni and Sharpe, 2019). Umbilical artery-derived perivascular stem cells (UCA-PSCs) are a kind of umbilical artery MSCs with higher proliferation and differentiation potentials than normal HUMSCs (Xu et al., 2017a). By adding UCA-PSCs containing GelMA solution to the MN tips, and antioxidant cerium oxide (CeO2) nanozyme containing GelMA solution to the back, Zhu et al. presented a novel flexible multifunctional biohybrid microneedle patch and administrated it in rat endometrium (Zhu et al., 2022). This GelMA based MN can remove excessive ROS and avoid the oxidative stress at the site of endometrial injury by release of CeO_2_ nanoparticles with multienzyme-like activity and deliver UCA-PSCs into injury tissue to resulted in highly promoted regeneration of smooth muscle and neovascularization.

**Hyaluronic acid (HA)** is a glycosaminoglycan that occurs naturally within the body. It is sourced from physiological origins and is noted for its compatibility with biological systems, lack of allergenic potential, anti-inflammatory characteristics, and ability to biodegrade (Nappi et al., 2007). The remarkable biocompatibility of HA, coupled with its capacity to suppress inflammation, positions it as a highly suitable option for applications in tissue regeneration, particularly in the context of endometrial reconstruction (Li et al., 2021; Ji et al., 2022). Chemical modifications on HA can help the auto-crosslinking between molecules and can significantly enhance its *in situ* retention time and MSC encapsulation efficiency, thereby optimizing their therapeutic potential in various medical applications. Studies have demonstrated that auto-crosslinked HA gel can effectively reduce the incidence and severity of IUAs when applied postoperatively (Luo et al., 2024; Sroussi et al., 2022). Greater prevention of recurrent IUAs in patients than control (Trinh et al., 2022; Xiao et al., 2015). Thus, commercial auto-crosslinked HA gels as a promising physical barrier, has been approved by China Food and Drug Administration (CFDA) as a medical device for clinical practice after hysteroscopic adhesiolysis to achieve improvement in histocompatibility and viscosity.

In the context of biocompatibility, Liu et al. evaluated the cytotoxicity of several MSC conventional carriers to SUSD2+ endometrium MSC (EnMSC), including HA-gel, collagen scaffolds, 2.5% crosslinked polyacrylic acid (cPAA), and a complex formulation of 15% poly (D, L-lactic acid-co-glycolic acid)-B-poly (ethylene glycol)-B-poly (D, L-lactic acid-co-glycolic acid) (PLGA-PEG-PLGA). Their findings indicated that among all these carriers, HA gel demonstrated remarkably high biocompatibility to EnMSCs (Liu et al., 2023). In the subsequent application of MSC therapy within a rat model designed to simulate endometrial mechanical injury, the intrauterine transplantation of EnMSCs loaded within the HA gel resulted in a significant enhancement of the endometrial thickness and an increase in the number of glands present in the tissue. Additionally, this innovative approach led to improved blood flow within the endometrium, a reduction in fibrotic regions, and a notable increase in pregnancy rates, highlighting the efficacy of HA gel as a carrier for EnMSC s in regenerative medicine (Liu et al., 2023). The dual repair effects of HU-MSCs loaded auto-crosslinked HA gel on endometrial damage and adhesion have been explored in both rat (Fan et al., 2024) and nonhuman primates (Wang et al., 2020) IUA model. The first nonhuman primate IUA model was established in rhesus monkeys by Wang and colleagues via open abdominal surgery. By treated the endometrium injured monkeys with HU-MSCs loaden commercial HA gel (Bioregen, Co., Ltd. China), notable dual repair effects: a reliable antiadhesion property and the promotion of endometrial regeneration were observed (Wang et al., 2020). As the first non-human primate MSC/HA gel utilized in the treatment of IUA, this groundbreaking advancement holds immense significance in the field of reproductive medicine, even though there remains a pressing need for enhancements in the method of administration to optimize its effectiveness and clinical application.

Chemically modified HA gel, or hyaluronic acid gel, represents an advanced form of hyaluronic acid that has undergone specific chemical alterations to enhance its properties and applications. This innovative gel is designed to improve its stability, viscosity, and bioavailability, making it particularly useful in various fields such as dermatology, ophthalmology, and regenerative medicine. For example, Hu and colleagues prepared injectable UC-MSCs-laden injectable auto-crosslinked hydrogels by mixing UC-MSCs suspended methylfuran-modified hyaluronan (HA-F) solution and maleimide-modified hyaluronan (HA-A) solution. The UCMSCs encapsulated into the 3D structure of the hydrogel can survive for 5 days after and help restore the morphology and functionality of the damaged endometrium by inducing cell proliferation, migration, angiogenesis (Hu et al., 2024). Furthermore, by mixing human UC-MSCs (5 × 10^6^ cells) suspending hydrazide-grafted gelatin (Gel-ADH) solution with oxidized hyaluronic acid (HA-CHO) solution rapidly, Zhang et al. prepared a novel human UC-MSCs s-loaded hydrogel for rat IUA therapy (Zhang et al., 2023). Lin et al. designed human placenta-derived MSCs (HP-MSCs) loaded HA gel, by encapsuling HP-MSCs in to a UV photo-crosslinked glycidyl methacrylate functionalized HA (GAM-HA) gel. After injected with the HP-MSCs loaded HA gel, the injured rat uterus achieved rapid recovery of endometrial, embryo implantation and live birth rates restore to normal level (Lin et al., 2022). All the purified ECM-related biomaterials discussed in this section, along with their applications in endometrial regeneration, have been summarized in Table 1.

**TABLE 1:** The purified extracellular matrix-related biomaterials utilized for MSC delivery in endometrium regeneration.

Biomaterials	Form	Modification	Models	Cell	Treatment	Ref
Collagen	Scaffolds	—	Rat endometrial injury model (ethanol perfusion)	Human AD-MSCs	Collagen scaffold seeding with human ADMSCs (1 × 10^6^)	Dai et al. (2023)
			Rat endometrium damage model with mechanical injury and LPS surgical suture)	Human MenSCs	0.05 mL per uterus (2 × 107 cells/mL with 1.0 cm × 0.5 cm scaffold	Hu et al. (2022a)
			Rat endometrium damage model with mechanical injury	Human UC-MSCs	collagen scaffold segment (2.5 cm × 0.5 cm) loaded with 5 × 10^5^ UC-MSCs	Xin et al. (2019)
				Human UC-MSCs	Collagen scaffold (1.5 × 0.5 cm) seeding with 3 × 10^6^ hUCMSC	Liu et al. (2020)
			Rat uterine damage model (resecting segment of the uterus horn, retaining the mesometrium)	Rat BM-MSCs	Collagen scaffold seeding with Rat BM-MSCs (5 × 10^5^ cells/cm^2^)	Ding et al. (2014)
				HESCs derived endometrium-like cells	Collagen scaffold (1.5 × 0.5 cm) seeding with 3 × 10^6^ hESCs-derived endometrium-like cells	Song et al. (2015)
				Human En-PSCs (overexpressing CYR61)	Collagen scaffold (1.5 × 0.5 cm) seeding with Human En-PSCs were sutured into injured area to replace the excised segment	Li et al. (2019b)
			Patients with secondary infertility or embryo transfer failure caused by recurrent IUA	Human UC-MSCs	human UC-MSCs (4.2 × 10^5^/cm^2^) were loaded on Collagen scaffold (4 cm × 6 cm) Clinic trial NO. NCT02313415	Cao et al. (2018)
			Patients with infertility and thin endometrium	Human UC-MSCs	human UC-MSCs (2 × 10^7^/mL) loaded on collagen scaffolds (4 cm × 6 cm) Clinic trial NO. NCT03724617	Zhang et al. (2021)
			Patients with infertility caused by thin endometrium or endometrial scarring	Human UC-MSCs	Not mentioned Clinic trial NO.NCT03592849	
			Patients with infertility caused by severe IUA	Autologous BM-MSCs	Not mentioned Clinic trial NO.NCT02204358	
	scaffold based endometrial patch	3D bioprinting endometrial patch with bioink: alginate, collagen, PF-127	Rat thin endometrium model with ethanol perfusion	Human AD-MSCs	AD-MSCs (5 × 10^5^) loaded patch were transplanted into the uterine cavity through small incision	Hong et al. (2023)
GelMA	scaffold	PH scaffolds bioprinting with GelMA/PEO	Rat IUA model with mechanical injury and LPS surgical suture	Rat AD-MSCs	ADSCs loaded PH scaffolds (2 × 10^5^ cells/mL)	Lu et al. (2023)
	hydrogel	ColMA/GelMA	Rat IUA model with mechanical injury	Human AMSCs	GelMA/ColMA hydrogel containing hAMSCs (1 × 10^7^ cells/mL) were 3D printed and placed into uterine cavity after construction of the IUA moldel	Feng et al. (2021)
	injectable hydrogel	GelMA/SerMA	Mice IUA model with ethanol perfusion	Human UC-MSCs	UC-MSC (1 × 10^6^ cells) loaded hydrogel were injected into uterus, then the uterus was exposed to 405 nm blue light for 30 s	Chen et al. (2023)
	Microneedles	Lactoferrin	Rat AS model with full-thickness resection of the uterus	Human En-ADVs spheroid	MN/En-ADV patches were covered onto the wounds to form close cavity	Li et al. (2022)
	Microneedles	CeO2 nanozyme	Rat AS model with full-thickness resection of the uterus	UCA-PSCs	MN/UCA-PSC patches were covered onto the wounds to form close cavity	Zhu et al. (2022)
Hyaluronic acid	hydrogel	—	Rat endometrial mechanical injury model	EnMSCS	2 × 106 EnMSCs and HA-GEL were aspirated with a 1-mL syringe and injected intrauterinely through endometrial injury incision	Liu et al. (2023)
		—	Rhesus monkeys IUA models with mechanical injury (intraperitoneal surgery)	Human UC-MSCs	UC-MSCs (1 ∼ 2 ×10^7^ cells) loaded commercial HA gel were immediately transplanted into the uterine cavity	Wang et al. (2020)
		HA-CHO/Gel-ADH	Rat IUA model with mechanical injury	human UC-MSCs	hUC-MSCs (5 × 10^6^ cells) loaded HA-CHO/Gel-ADH hydrogel were injured into uterus	Zhang et al. (2023)
		HA-F/HA-A	Rat endometrial chemical injury model with ethanol perfusion	Human UC-MSCs	UC-MSCs loaded hydrogel (1 × 10^7^ cells/mL) 100 μl were injected	Hu et al. (2024)
		GAM-HA	Mice chemical injury model with ethanol perfusion	HP-MSCs	intrauterine instillation of 25 μL HP-MSCs loaded gel (2 × 10^5^ HP-MSCs per/uterus)	Lin et al. (2022)

Abbreviation: LPS, lipopolysaccharide; PEO, polyethylene oxide; GelMA, gelatin methacryloyl; ColMA, methacrylated collagen; SerMA, methacrylate sericin; CeO2, cerium oxide; HA-CHO, oxidized hyaluronic acid; Gel-ADH, hydrazide-grafted gelatin; HA-F, methylfuran-modified hyaluronan; HA-A, maleimide-modified hyaluronan; GAM-HA, glycidyl methacrylate functionalized HA; AD-MSCs, adipose-derived mesenchymal stem cells; AMSCs, amniotic MSCs; BM-MSCs, bone marrow mesenchymal stem cells; HESC, human embryonic stem cells; MenSCs, menstrual blood stem cells; UC-MSCs, human umbilical cord mesenchymal stem cells; En-ADVs, endometrium-derived adventitial cells; UCA-PSCs, Umbilical artery-derived perivascular stem cells; EnMSCs, endometrium mesenchymal stem cells; En-PSCs, endometrial perivascular cells; HP-MSCs, human placenta-derived mesenchymal stem cells.

#### 4.1.3 Beyond cell delivery-natural materials used in 3D bio-printing

Natural materials used in general tissue engineering presents significant opportunities for the regeneration of endometrium. However, challenges surrounding personalization and precision in these processes persist. 3D bioprinting technology technique enables the precise placement of MSCs within biocompatible hydrogels, creating scaffolds with specific microarchitectures that support cell adhesion, proliferation, and differentiation and facilitates subsequent endometrial repair (Hong et al., 2023; Lu et al., 2023). By creating highly precise and biomimetic constructs, 3D bioprinting has revolutionized the field of tissue engineering, enabling the fabrication of complex, multilayered artificial endometrial constructs through meticulous control over the spatial arrangement of various cells and bioactive components.

In a groundbreaking study, Park et al. developed a 3D stem cell-laden artificial endometrium utilizing a combination of collagen I, hyaluronic acid, and diverse endometrial cells, which remarkably maintained their molecular characteristics, thereby closely mimicking the natural environment of the endometrium. Following rat uterine ablation with 2% TCA, the transplantation of this innovative artificial endometrium not only alleviated injuries but also led to successful pregnancies, showcasing its potential therapeutic benefits. However, this approach is not without its limitations; it features a simplified three-layer structure, exhibits an altered cell distribution compared to the natural endometrium, and poses the risk of potential leakage from the uterine cavity, which necessitates the use of sutures that may inadvertently cause damage and limit its clinical applicability (Park et al., 2021). Based on this investigation, Park et al. further advanced their research by creating an adaptable, Lego-inspired multi-module endometrial tissue system that facilitates a scalable, patient-specific tissue environment for regeneration. This is achieved through the injection of collagen, hyaluronic acid, and cells into a precisely designed 3D bio-printed mold (Park et al., 2023). Although this innovative construct has only been evaluated in a skin defect model thus far, the prospective application in endometrial repair remains highly encouraging and holds significant promise.

In summary, ECM-related natural materials hold significant importance and offer a wide range of applications in the context of MSC-based endometrial regeneration. Despite of the benefits mentioned above, natural materials also have certain limitations. For instance, as derived from biological sources, the natural materials may suffer from batch-to-batch variability and result in inconsistent treatment outcomes. Additionally, the different biological origin of natural materials may lead to potential immunogenicity, particularly the non-autologous materials. Conversely, synthetic biomaterials offer significant advantages, including their tunability and consistency. Furthermore, synthetic biomaterials can be manufactured on a large scale with high reproducibility, ensuring consistent quality in clinical applications. Therefore, a large number of synthetic materials are currently being used for MSC therapy.

### 4.2 Synthetic materials

As mentioned above, synthetic materials are ease for large-scale manufacturing and adjustable biological characteristics for specific applications. Consequently, the synthetic substances with excellent biocompatibility such as Poly (polyethylene glycol citrate-co-N-isopropylacrylamide) (PPCN), Poly-(glycerol sebacate) (PGS), polycaprolactone (PCL) and Pluronic F-127 (PF-127) are frequently employed in MSC-based endometrial regeneration.

**PPCN** is a thermos-responsive biomaterial which undergoes the reversible phase change from liquid to solid at 37°C (Yang et al., 2014). It has good biocompatibility and controllable degradability and is often used in cell delivery and tissue engineering. However, with poor mechanical robustness and inflammatory degradation, the application of PPCN is limited. By mixing PPCN with gelatin, PPCNg is formed to increase cell adhesion and support cell survival and implantation (Zhu et al., 2016). A recent study has shown that PPCNg can improve the nutrient supply of transplanted MSCs at the injured site and provide further growth space (Xiao et al., 2021). Intrauterine injection of human AD-MSCs loaded PPCNg hydrogel can facilitate the regeneration of injured endometrium by improving utilization rates of human AD-MSCs at injured site and eventually restore reproductive capacity in rat IUA model (Huang et al., 2022b). As PPCNg can enhance vaginal colonization of MSCs, Huang investigated the effects of vaginal and intrauterine injections of human AD-MSCs loaded PPCNg hydrogel on endometrial repair in a rat model of IUA. The findings from this study revealed that the vaginal administration of human AD-MSCs resulted in a notable enhancement in endometrial thickness as well as an increase in the number of glands present within the endometrium. Interestingly, there was no significant difference observed when these results were compared to those from the intrauterine injection group. This suggests that the mechanism by which MSCs contribute to the rehabilitation of the endometrium primarily operates through paracrine signaling pathways, rather than relying on trans-differentiation processes (Huang et al., 2024b).

**PGS** exemplifies synthetic bio-elastomers. It effectively withstands and rebounds from deformations in soft tissues under dynamic conditions, while also minimizing discomfort to adjacent structures. Therefore, it can be effectively used in larger tissues, such as for endometrial repair. Furthermore, PGS porous scaffolds have proven to be effective carriers for various cell types, particularly mesenchymal stem cells (Xu et al., 2017b). Xiao et al. designed a BM-MSCs loaded PGS scaffold and explored its endometrial repair capacity in rat IUA model. In contrast to direct BMSC intrauterine injection, as well as poly-(lactic-co-glycolic acid) (PLGA) and collagen scaffolds, the transplantation of the PGS scaffold markedly extends the retention duration of BMSCs in a wounded rat uterus model. The transplantation of BMSC-laden PGS scaffold effectively enhances the differentiation rate of BMSCs and increases the concentration of growth factors in the damaged endometrium (Xiao et al., 2019).

**PF-127**, also known as Poloxamer 407, is a widely used FDA proved triblock copolymer in biomedical applications with unique thermo-responsive properties, biocompatibility, and ability to form hydrogels at body temperature (Cortiella et al., 2006; Vashi et al., 2008). The thermo-reversible gelation of PF-127 renders it an optimal medium for cell encapsulation to facilitate cell adhesion as a scaffold. Although PF-127 is generally considered biocompatible, studies have reported dose-dependent cytotoxic effects on various cell types. At higher concentrations, PF-127 can disrupt cellular membranes, leading to cell lysis and death (Alvarado-Gomez et al., 2018; Matthew et al., 2002). This is particularly concerning for sensitive MSCs used in tissue engineering and regenerative medicine applications.

By adding potent reducing agent Vitamin C (Chandrasekaran et al., 2017) to alleviate the cytotoxic effect of PF-127, Yang et al. successfully achieved endometrial regeneration in rat IUA models with intrauterine transplantation of PF-127-encapsulated BMSCs (Yang et al., 2017). Their results suggested that vitamin C added PF-127 can foster the survival of encapsulated BMSCs *in vitro* through an IL-10-independent mechanism. Upon transplantation of this combination *in vivo*, the endometrium displayed significant rejuvenation, marked by a thicker endometrial membrane that was enriched with additional glands and demonstrated a substantial decrease in fibrotic areas, signifying a successful regenerative process.

In the intricate field of uterine scar treatment in rats, the PF127 hydrogel emerges as a promising candidate, showcasing significant potential for a variety of future applications. In a pioneering study conducted by Hu et al., the researchers first engineered an innovative injectable thermosensitive hydrogel utilizing aldehyde-functionalized PF127 (PF127-CHO) in conjunction with adipic dihydrazide-modified hyaluronic acid (AHA), creating a novel hydrogel formulation. Following this, the team prepared asiaticoside-encapsulated poly (DL-lactide-co-glycolide) (PLGA) microspheres, referred to as AMs, with the primary aim of enhancing the release stability and longevity of the anti-inflammatory compound asiaticoside. This advancement led to a notable increase in the *in situ* absorption of asiaticoside, thereby improving its therapeutic efficacy. Ultimately, by incorporating umbilical cord mesenchymal stem cells (UCMSCs) and the previously developed AMs into the PF127-CHO/AHA hydrogel, they successfully developed a composite hydrogel designated as PF127-CHO/AHA/AMs/UCMSCs. This innovative hydrogel was then administered via intrauterine injection into a rat uterine scar model. Remarkably, after the intrauterine administration, this hydrogel demonstrated its effectiveness by significantly facilitating the regeneration of glandular structures, reducing the extent of endometrial fibrosis, and restoring the morphological integrity of the uterine cavity, thereby highlighting its potential as a therapeutic agent in the treatment of uterine scars (Hu et al., 2022b).

**PCL**, as United States Food and Drug Administration (FDA)-approved poly (α-esters), have been reported to effectively serve as non-toxic substrates for the *ex vivo* propagation of MSCs (Sekuła et al., 2017). Due to its remarkable flexibility and impressive ductility, which significantly contribute to the formation of uniform and consistent nanofibers, PCL is frequently utilized in the production of cell loading electrospun (Mondal et al., 2016), which can effectively mimic the extracellular matrix, providing a suitable microenvironment for MSCs growth and function in endometrial repair (Kim et al., 2012). However, various drawbacks of PCL, such as inadequate mechanical properties, hydrophilic characteristics, sluggish degradation rates, and diminished cell affinity have greatly limited the application of PC Las cell scaffolds.

Silk fibroin (SF), a natural protein renowned for its exceptional mechanical properties, biodegradability, biocompatibility, and bio-resorbability, has garnered considerable interest for tissue engineering applications (Sun et al., 2021; Liu et al., 2022). By adding the appropriate amount of SF into PCL, Zhou et al. successfully prepared SF/PCL electrospun fibers, which ultimately results in a composite material that exhibits remarkable mechanical properties and enhanced bioactivity (Zhou et al., 2023). Loaded with AD-MSCs, the AD-MSCs-SF/PCL eletrospun were formulated and effectively augmented the *in situ* proliferation of transplanted AD-MSCs. The findings indicated that the AD-MSCs-SF/PCL system (2.5 × 0.5 cm^2^, containing 1 × 10^6^ AD-MSCs, per rat, for one uterine cavity) could restore morphology, stimulate gland regeneration and angiogenesis by elevating CD31 expression, and reverse endometrial fibrosis by diminishing TGF-β/Smad expression and also system remodel the unique immune microenvironment (Zhou et al., 2023).

In general, the diversity of synthetic materials provides more choices for MSC-based endometrial reconstruction. A large proportion of investigators are trying to use FDA-approved materials as MSC carriers for their significant advantages in both regulatory and clinical applications. All the synthetic materials discussed in this section, along with their applications in endometrial regeneration, have been summarized in Table 2. These materials have been rigorously evaluated for safety and can reduce the risk associated with immune rejection or adverse reactions during clinical use. Utilizing FDA-approved materials can simplify the clinical translation of new treatments and reduce the time and cost required for preclinical testing. In summary, natural materials excel in providing a biomimetic environment that enhances MSC functionality but may face challenges in consistency and mechanical properties. Synthetic materials, on the other hand, offer precise control and reproducibility, with the potential to be customized for specific therapeutic applications, but may require additional modifications to match the bioactivity of natural materials.

**TABLE 2:** The synthetic biomaterials utilized for MSC delivery in endometrium regeneration.

Biomaterials	Advantages	Limitations	Form	Combination	Models	Cell	Treatment	Ref
PPCN	Thermos-responsive good biocompatibility and controllable degradability	Poor mechanical robustness and inflammatory degradation	Hydrogel	Gelatin (PPCNg)	Rat IUA model (ethanol perfusion)	Human AMSCs	1 × 10^7^ hAMSCs resuspended in 100 μL PPCNg, injected into uterine cavity Or vagina	Huang et al. (2022b) and Huang et al. (2024b)
PGS	Bio-elastomers with modulus similar to the one of native rat uterus tissue	Limited Mechanical Strength, Fast Degradation Rate	Scaffoldd	—	Rat IUA model (mechanical damage)	Rat BM-MSCs	BM-MSCs (6 ∼ 7 × 10^5^ cells cm^−2^) loaded on 1.5 cm × 0.5 cm^2^ PGS scaffold were injected into uterine cavity	Xiao et al. (2019)
PF-127	Thermo-responsive properties, biocompatibility	Dose-dependent cytotoxic effects	Hydrogel	Vitamin C (PF-127 plus Vc gel)	Rat IUA model (mechanical damage)	Rat BM-MSCs	8 × 10^5^ BM-MSCs loaded in PF-127 plus Vc gel, injected into uterine cavity	Yang et al. (2017)
				AHA (F127-CHO/AHA gel)	Rat uterine scar model (mechanical damage)	Human UC-MSCs	1 × 10^6^ UC-MSCs loaded in F127-CHO/AHA gel, injected into uterine cavity	Hu et al. (2022b)
PCL	Low toxicity, flexibility, ductility	Poor mechanical properties, hydrophilicity and degradation	Electrospun	silk fibroin (SF/PCL fibers)	Rat IUA model (mechanical damage)	AD-MSCs	2.5 × 0.5 cm^2^, scaffold with 1 × 10^6^ AD-MSCs, per rat uterine cavity	Zhou et al. (2023)

Abbreviation: AMSCs, amniotic mesenchymal stem cells; AD-MSCs, adipose-derived mesenchymal stem cells; BM-MSCs, bone marrow mesenchymal stem cells; UC-MSCs, human umbilical cord mesenchymal stem cells; PPCN, Poly (polyethylene glycol citrate-co-N-isopropylacrylamide); PGS, Poly-(glycerol sebacate); PCL, polycaprolactone; PF-127, Pluronic F-127; F127-CHO, aldehyde-functionalized Pluronic F127; AHA, adipic dihydrazide-modified hyaluronic acid.

## 5 Biomaterials used in MSC based cell-free therapies of endometrial regeneration

**FIGURE 3:**
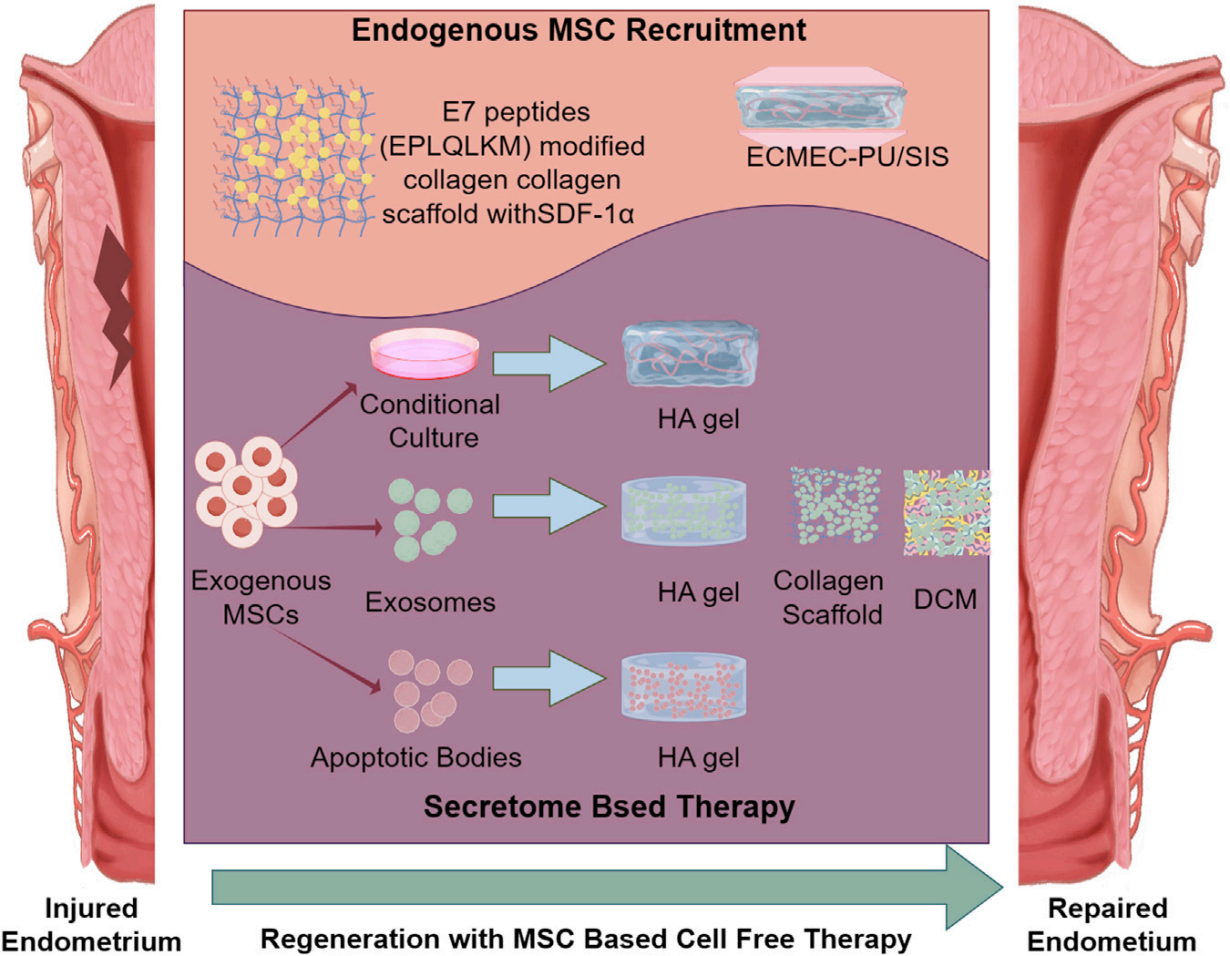
Biomaterials used in MSC based cell-free therapies of endometrial regeneration.

As discussed in this review, combined with biomaterials, MSCs therapy has achieved significant advancements in the realm of endometrial repair, demonstrating its capacity to revolutionize treatment options. Nevertheless, challenges in clinical setting, such as immunogenicity, potential tumorigenicity, teratogenicity, and the inconvenient in transport and storage have greatly restricted the application of MSC therapies (Heslop et al., 2015; Benor et al., 2020). Consequently, several MSC based cell-free therapies have been developed for endometrial regeneration, primarily encompassing the harness of endogenous MSC and the employment of secretome such as MSC-derived exosomes and apoptotic bodies both of which have been shown in Figure 3.

### 5.1 BioMaterial based endogenous MSC recruitment

In endometrial regeneration, biomaterials can not only provide essential structural support but also delivery bioactive factors to favorable MSCs attachment, proliferation, and differentiation. By added or modified with specific bio-factors to attract endogenous MSCs, some innovative biomaterials have been applied in cell-free endometrial regeneration. These strategic approaches can not only reduce the immune risk of exogenous MSCs transplantation, which is also complex and costly, but also promote a more organic and natural regeneration process.

Small intestinal submucosa (SIS) is a bioderived material that has garnered significant attention in the field of regenerative medicine. In a prior study conducted by Zhao et al., endometrial epithelial cells (ECs) derived ECM were seeding on SIS or Polyurethane/SIS (PU/SIS) scaffold to prepare tissue-specific ECM modified scaffold, ECM_EC_-SIS and ECM_EC_-PU/SIS. Then the ECM-SPS were prepared by sticking ECM_EC_-SIS onto ECM_EC_-PU/SIS. Their discoveries in rat IUA model revealed that the EC derived ECM modification on SPS possessed the remarkable ability to induce a directed recruitment and differentiation of endogenous MSC. Moreover, the compact intimal layer of SPS can serve as physical barrier to prevent IUA (Zhao et al., 2023). Thereby, we believe this novel ECM-modifying scaffold may open new avenues for advancements in endometrial regeneration. Nevertheless, concerning of the complex function of ECM, the speculation of Zhao derived from literature reviews and limited experimental data can’t demonstrate the molecular mechanism of the recruitment of endogenous MSC derived by ECM-SPS, nor is it evident that which compose of ECM has assumed a significant role, leaving a gap in our understanding of the intricate interactions at play within the biological context.

For the biomaterials that do not inherently attract endogenous MSCs, modification with molecules that draw MSCs is an efficient method to harness endogenous MSCs. Previous research has demonstrated that E7 peptides (EPLQLKM) modified collagen can selectively capture MSCs *in vitro* (Zheng et al., 2018). Furthermore, stromal-derived factor-1 alpha (SDF-1α) is a powerful chemoattractant for MSCs via C-X-C chemokine receptor type 4 (CXCR4) (Wenbo et al., 2020). Given the short half-life of SDF-1α protein, strategies of SDF-1α association with biomaterials have also been developed. Xin et al., developed an acellular scaffold named CES by combining SDF-1*α* with E7-modified collagen scaffold which successfully harnessed the innate regenerative potential of the body and enabled near-complete endometrium regeneration and fertility restoration in both rat endometrium acute damage model and IUA model (Xin et al., 2022a).

As a result, by leveraging the body’s intrinsic regenerative capabilities, these approaches presented a promising tactic for the restoration of the endometrium, ultimately contributing to better outcomes in regenerative medicine and tissue repair. These strategies not only facilitated the regeneration of damaged tissues but also minimized the need for cell-based therapies, which often present challenges related to immune and ethical concerns.

### 5.2 Biomaterials used in MSC secretome based therapy of endometrial regeneration

**MSC secretome**, the collection of bioactive cellular content secreted by MSCs, including MSC-derived exosomes, apoptotic bodies, and various bioactive factors, who plays crucial roles in mediating the regenerative and paracrine effects of MSCs on surrounding tissues. As paracrine signaling pathways have been shown to play a crucial role in the ability of MSCs to facilitate endometrial regeneration (Huang et al., 2024b). *In vitro* studies have demonstrated that the secretome of MSC can enhance cell proliferation and migration by activation of PI3K/Akt or FAK/ERK1/2 signaling cascades (Park et al., 2018). Thus, MSC secretome and its contents have been harnessed in endometrial regeneration too. The MSC secretome have shown improved safety profile and enhanced stability over traditional MSC transplantation which can make secretome therapy a more clinically translatable strategy.

**MSC derived conditional culture** is a remarkably complex and multifaceted solution that rich in secretome and can profoundly impact cellular behavior, enhance tissue regeneration. Liu et al. infused crosslinked methacrylate HA gel with MSC derived conditional culture to form MSC-Sec gel, establishing a sustained secretome release system that effectively repaired endometrial injury in rats and facilitated viable pregnancy. This therapeutic platform leverages paracrine signaling proteins akin to the efficacy of cell therapy, while addressing the challenges associated with live stem cells (Liu et al., 2019). It is particularly noteworthy that, unlike the laparotomy method employed in this animal study, which involves a more invasive surgical approach, clinical treatment for IUA is generally administered vaginally to enhance patient comfort and therapeutic effectiveness. Therefore, the administration of MSC-Sec gel necessitates further comprehensive studies to evaluate its safety, efficacy, and potential for clinical use in human patients.

**Exosomes**, as nano-sized extracellular vesicles derived from MSCs, are particularly significant within the secretome. Recently, the exosomes derived from stem cells have shown its ability to induce immunomodulation, cell proliferation with low immunogenicity, positioning them as a promising alternative to traditional cell therapies (Brock et al., 2019; Lin et al., 2023), especially in endometrial regeneration (Wang et al., 2023). However, there are still limitations regarding the application of exosomes, as they can be quickly removed from injured sit in a short time. The application of biomaterials can not only optimize the availability of the bioactive molecules contained within the exosomes but also provide essential structural support for endometrial repair, thereby enhancing the overall effectiveness of therapeutic interventions and promoting a more favorable environment for tissue repair and regeneration. By integrating exosomes with biomaterials, a controlled and sustained release of exosomes can be achieved.

Xin et al. designed a construct of UC-MSC exosomes loaded collagen scaffold (CS/Exos). The CS/Exos transplantation promoted endometrium regeneration, collagen remodeling, estrogen receptor α/progesterone receptor expression and resulted in fertility restore in rat endometrium-damage model by facilitating CD163+ M2 macrophage polarization (Xin et al., 2020). By pretreating HUMSC with transforming growth factor-β1 (TGF-β1), Zhang et al. acquired high-quality HUMSC-exosomes and combined the exosomes with commercial HA gel (from BioRegen Biomedical, Changzhou, China) to enhance the stability of the exosome structure and extend the efficacy of the exosomes within the uterine cavity (Zhang et al., 2022b). The exosome loaded HA gel successfully promote pregnancy rate in rat AS model with thin endometrium.

Jin et al. successfully generated porcine dermis derived DEM and subsequently composited it with AD-MSCs derived exosomes to form a novel construct known as ECM@AD-MSC-exos. This innovative approach successfully achieved a sustained release of functional exosomes while simultaneously providing a supportive scaffolding layer on the surface of the injured endometrium which finally leading to facilitate the regeneration of a functional endometrium and preserved fertility (Jin et al., 2023).

**Apoptotic bodies (ABs)** are membrane-bound vesicles that are formed during cell apoptosis, containing complex cellular contents which can contribute to tissue integrity and function (Xu et al., 2019). The *in situ* survival of transplanted MSC has emerged as a significant obstacle that hinders the effective treatment of MSC in tissue regeneration, posing challenges for medical professionals and researchers alike. Evidences have shown that ABs played critical roles in compensatory tissue regeneration (Ma et al., 2020). However, directly injecting antibodies into the uterine cavity does not guarantee their effective retention. This can lead to low retention rates and unclear therapeutic outcomes, indicating that this delivery strategy is inadequate. Xin et al. incorporated human MSCs derived ABs into a HA gel to yield an AB-laden HA gel. In this hydrogel the ABs exerted therapeutic effects, whereas the HA promoted the retention of ABs at the injury site, simultaneously functioning as a physical barrier to prevent IUA. Thus the transplantation of this AB-laden HA gel successfully achieved cell-free endometrial regeneration and fertility restoration in both murine acute endometrial damage model and rat IUA model, showcasing its potential as a groundbreaking therapeutic approach in reproductive medicine (Xin et al., 2022b).

## 6 Discussion

In conclusion, innovative biomaterials represent a promising frontier in regenerative medicine, particularly for MSC-based endometrial reconstruction. Their ability to modulate the cellular microenvironment and enhance tissue regeneration has significant potential for improving therapeutic outcomes. During the intricate process of endometrial repair, biomaterials play a crucial role in creating a favorable microenvironment that benefits both MSCs and immune cells. Certain bioactive materials, particularly decellularized extracellular matrix, are known to actively regulate the immune response through the release of soluble factors. This sophisticated modulation not only enhances the survival and functional capabilities of transplanted MSCs but also significantly promotes the repair of damaged endometrial tissue by facilitating immune regulation. A comprehensive understanding of these immunomodulatory properties, along with the ability to harness them effectively, is essential for improving therapeutic outcomes in the complex field of endometrial reconstruction.

The translation of biomaterial-based therapies from the laboratory to clinical practice involves several regulatory challenges and critical considerations. One major concern is the immunogenicity associated with natural biomaterials, especially those derived from non-autologous sources, which may trigger adverse immune reactions. In contrast, synthetic biomaterials offer higher reproducibility and consistent quality, making them more favorable for clinical applications. Standardization of biomaterial production is essential to ensure uniformity in composition and performance across batches, addressing variability that could compromise therapeutic efficacy. Safety is another pivotal aspect, requiring thorough preclinical testing to evaluate biocompatibility, degradation profiles, and potential toxicity. Regulatory approval processes further demand robust clinical data to demonstrate efficacy and safety, often involving multiple phases of trials. Interestingly, our review found that many researchers have opted to use FDA-approved materials in MSC-based endometrial repair therapies. This approach likely stems from the dual benefits of ensuring safety and expediting the regulatory approval process, thereby facilitating quicker clinical translation.

Furthermore, MSCs themselves can be considered as active biomaterials too. MSC-derived spheroids and cell sheets have been increasingly recognized and utilized as promising therapeutic biomaterials for the purpose of endometrial reconstruction, offering innovative solutions to enhance tissue regeneration and repair (Sun et al., 2018; Ji et al., 2020). Moreover, enhancing our understanding of the endometrial microenvironment can help creating more effective artificial endometrial models and accelerate the advancement of endometrial reconstruction therapies. MSC-derived secretomes, such as conditioned media, exosomes, and apoptotic bodies are attracting more attention to mitigate the potential drawbacks associated with direct cell therapies while harnessing their regenerative potential more efficiently.

Ultimately, the application of biomaterials for both MSC based cell and cell-free delivery in endometrial repair holds significant promise for improving treatment outcomes and advancing therapeutic strategies in women’s reproductive health. The integration of biomaterials in endometrial repair strategies marks a significant advancement in addressing reproductive health issues, offering a hopeful avenue for enhanced quality of life for affected individuals.
